# Supplementary material for: The Nanostructure of Alkyl-Sulfonate Ionic Liquids: Two 1-Alkyl-3-methylimidazolium Alkyl-Sulfonate Homologous Series
Source: Molecules. 2023 Feb 23;28(5):2094. doi: 10.3390/molecules28052094 (PMC10004415; doi:10.3390/molecules28052094)
Supplement: Supplementary file 1 [file molecules-28-02094-s001.zip › molecules-2228055-supplementary.pdf]

Supplementary Material (SM) for:

The Nanostructure of Alkyl-Sulfonate Ionic Liquids: Two 1-Alkyl-3-methylimidazolium Alkyl-Sulfonate Homologous Series

Hugo Marques, José Nuno Canongia Lopes, Adilson Alves de Freitas *, Karina Shimizu *

Centro de Química Estrutural, Institute of Molecular Sciences, Instituto Superior Técnico, Universidade de Lisboa, Av. Rovisco Pais 1049 001 Lisboa, Portugal

* *E-mail: adilsondefreitas@tecnico.ulisboa.pt (AAF); karina.shimizu@tecnico.ulisboa.pt (KS)*

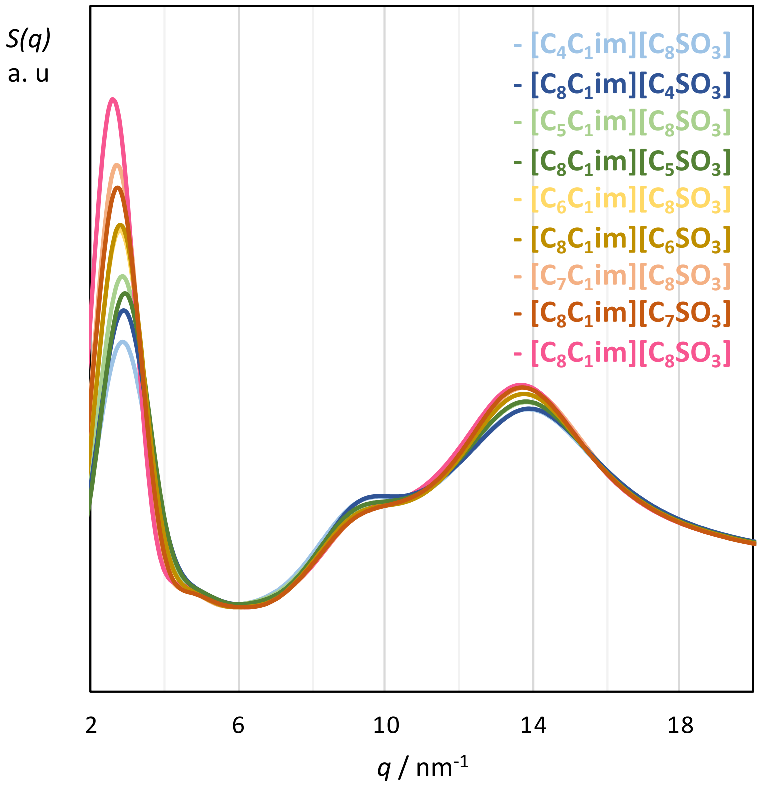

Figure S1: Structure factor functions, *S(q)*, for the $[C_nC_1im][C_8SO_3]$ and $[C_8C_1im][C_mSO_3]$ homologous series.

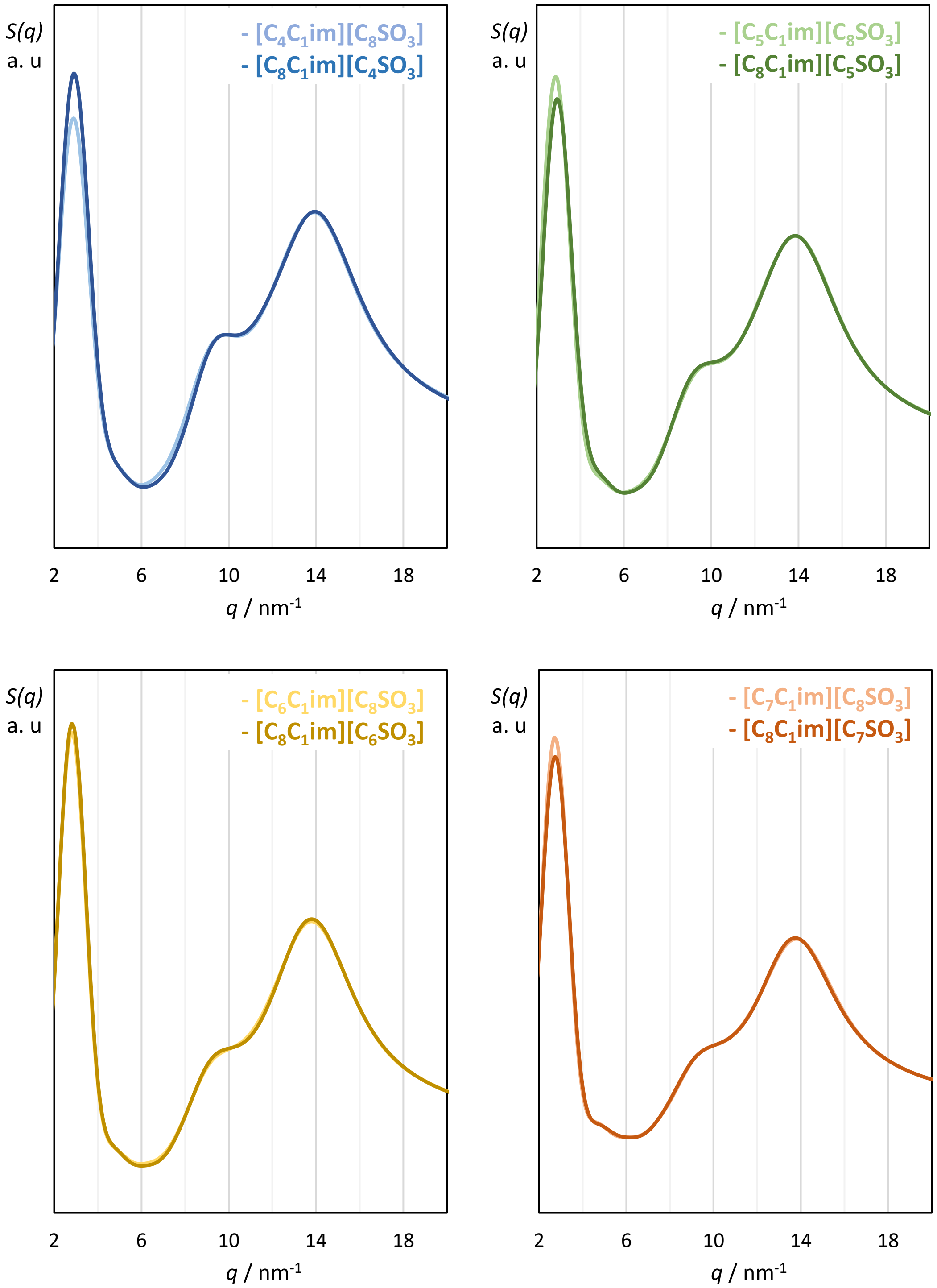

Figure S2: Structure factor functions for C_4 to C_7 analogue ILs.

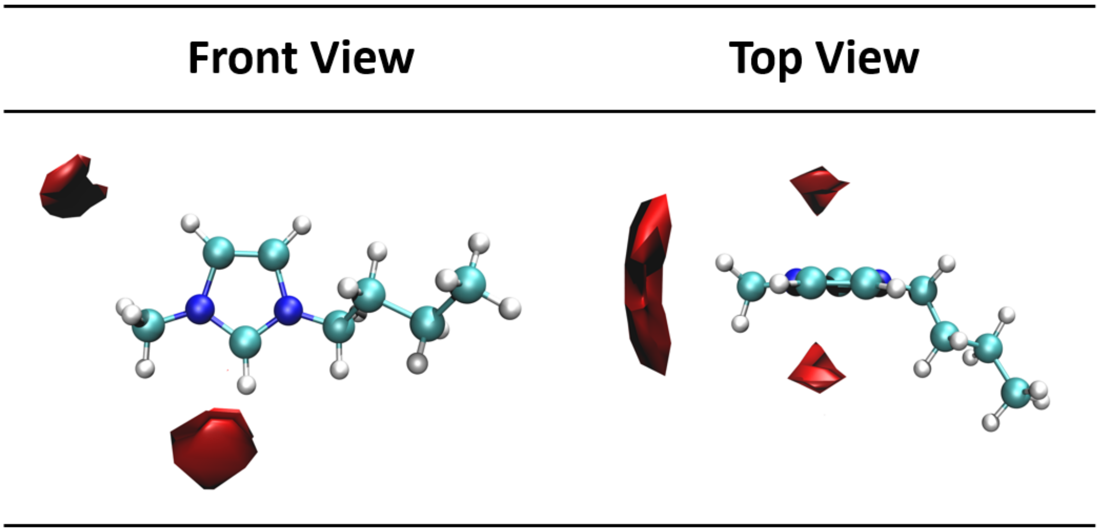

Figure S3: Selected spatial distribution functions around the imidazolium cation ring in $[C_4C_1im][C_8SO_3]$ IL, calculated from MD trajectories. The red colour represents the O atoms of the anion. The isosurface value corresponds to 70% of the maximum number density.

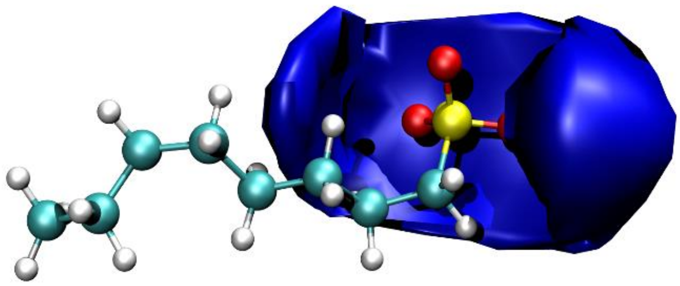

Figure S4: Selected spatial distribution functions around the $–SO_3^-$ group in $[C_4C_1im][C_8SO_3]$ IL, calculated from MD trajectories. The blue colour represents the N atoms of the imidazolium cation ring. The isosurface value is 70% of the maximum number density.

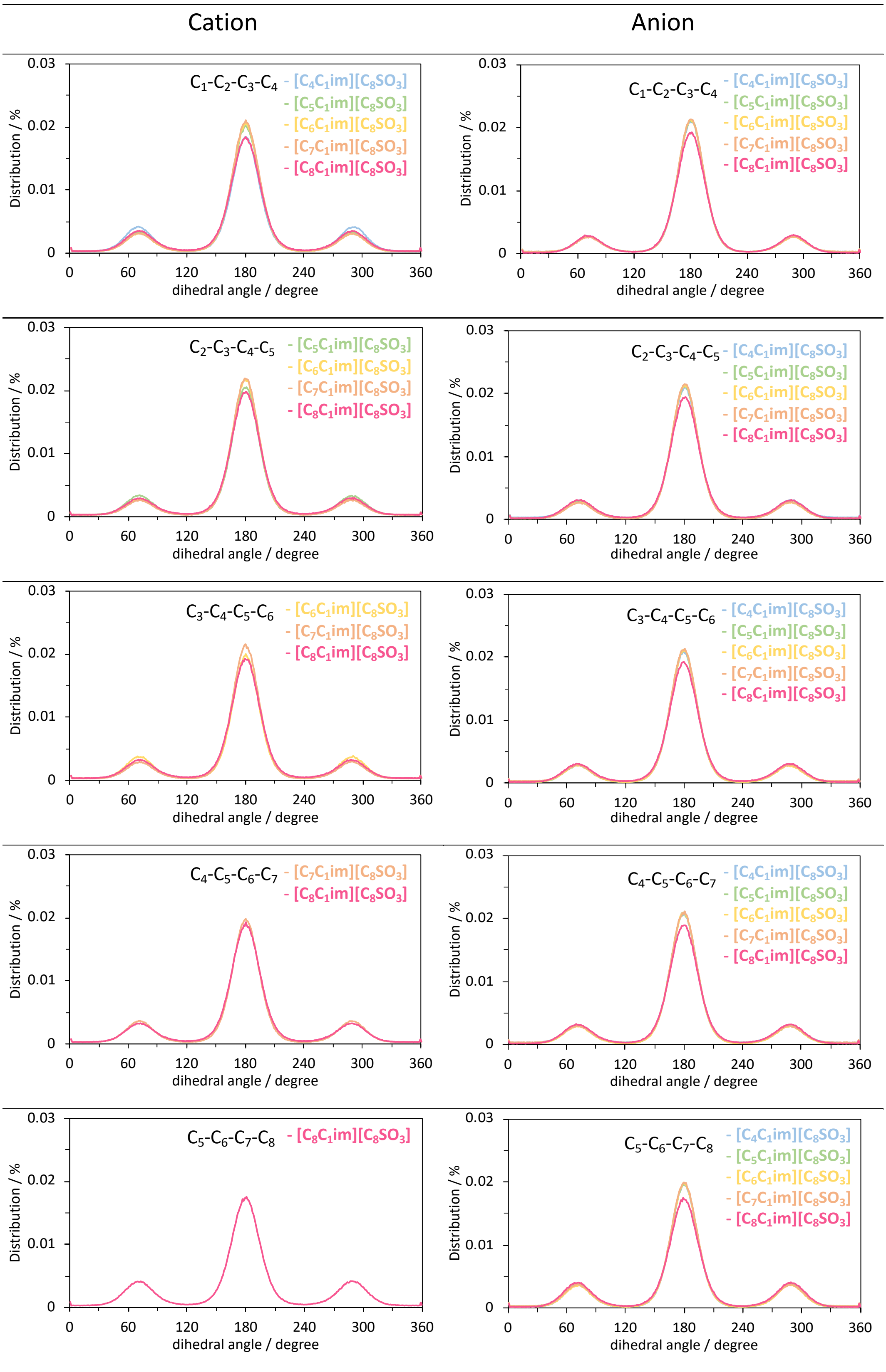

Figure S5: Probability distribution functions for dihedral angles of the alkyl tails in $[C_nC_1im][C_8SO_3]$ ionic liquids.

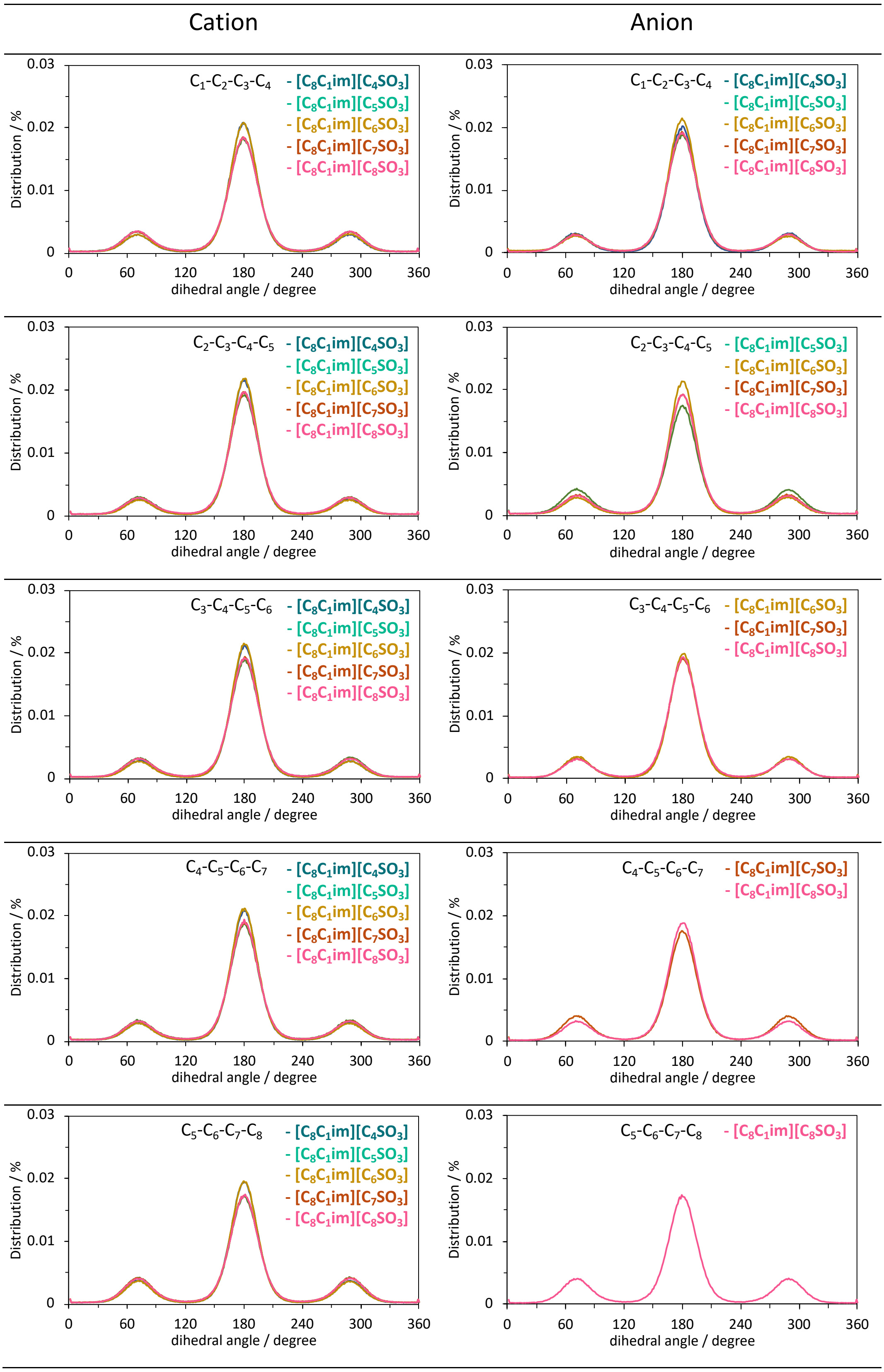

Figure S6: Probability distribution functions for dihedral angles of the alkyl tails in $[C_8C_1im][C_mSO_3]$ ionic liquids.
